# Z-Ala–Ile-OH, a dipeptide building block suitable for the formation of ortho­rhom­bic microtubes

**DOI:** 10.1107/S2053229623004849

**Published:** 2023-06-22

**Authors:** Renate Gessmann, Isabel Garcia-Saez, Georgios Simatos, Anna Mitraki

**Affiliations:** a IMBB/FORTH, N. Plastiras 100, 70013 Heraklion, Greece; b Université Grenoble Alpes, CNRS, CEA, Institut de Biologie Structurale (IBS), 38000 Grenoble, France; c IESL/FORTH, N. Plastiras 100, 70013 Heraklion, Greece; d Department of Materials Science and Technology, University of Crete, PO Box 2208, 71409 Heraklion, Greece; University of Oxford, United Kingdom

**Keywords:** chiral peptide, hydrogen bonding, microtube, Z-protection group, alanine, isoleucine, crystal structure

## Abstract

Self-assembling dipeptides have emerged in the last two decades as promising building blocks for the development of novel biomaterials. Among the various classes of dipeptides, aromatic dipeptides and especially di­phenyl­alanine (Phe–Phe), which forms hexa­gonal nanotubes, have been the most extensively studied. However, aliphatic peptides or mixed aromatic–aliphatic dipeptides seem just as promising, exhibiting various structures ranging from amyloid fibrils to microtubes. Herein we report the single-crystal structure of an aliphatic dipeptide, alanine–isoleucine (Ala–Ile), C_17_H_24_N_2_O_5_, protected with a benzyl­oxycarbonyl (Z) group at the N-terminus. The protected dipeptide crystallizes in the ortho­rhom­bic space group *P*2_1_2_1_2_1_ and forms hollow microtubes with ortho­rhom­bic symmetry upon evaporation on glass surfaces, as shown by field emission scanning electron microscopy (FESEM). These findings provide an increased understanding of the correlation between the single-crystal structure of the peptide building block and its self-assembly mechanism, and expand the library of available building blocks for microtechnological applications.

## Abstract 2

The aliphatic dipeptide alanine–isoleucine (Ala–Ile), protected with a benzyl­oxycarbonyl (Z) group at the N-terminus, forms hollow microtubes with ortho­rhom­bic symmetry upon evaporation on glass surfaces, as shown by field emission scanning electron microscopy (FESEM). These findings provide an increased understanding of the correlation between the single-crystal structure of the peptide building block and its self-assembly mechanism, and expand the library of available building blocks for microtechnological applications.

## Introduction

Self-assembling peptides tend to undergo spontaneous as­sem­bling into ordered biocom­patible nano- to microstruc­tures under mild conditions through weak, yet powerful, secondary or noncovalent inter­actions.

Due to the chemical variety provided by the 20 standard amino acids acting as structural building blocks, the resulting supra­molecular structures range from nanofibers and rods to spheres and ribbons (Gilead & Gazit, 2005 ▸). Studying the properties of self-organization has led to an increasing number of applications in various fields, such as biomedicine, bio/microtechnology and materials science (Adler-Abramovich & Gazit, 2014 ▸). In order to decipher the mechanisms behind self-assembly, one must link the effect of the solvent and conditions of the system (Mason *et al.*, 2014 ▸; Rissanou *et al.*, 2013 ▸), as well as the nature of the peptide (termini, side chains), order and types of inter­actions, to the obtained morphologies (Tao *et al.*, 2016 ▸). As such, the challenging task of correlating both the nature and outcome of the self-assembly process to the information encoded in each mol­ecular building block requires crystal structure information at the atomic level (Görbitz, 2001 ▸, 2003 ▸, 2007 ▸, 2010 ▸, 2018 ▸). Among the various kinds of peptides, aromatic peptides are the most extensively studied; the reason lies in the conjugated π-electron system contained in aromatic dipeptides that stabilizes π–π* stacking inter­actions and promotes self-assembly, while hydrogen-bond formation also constitutes a strong driving force for the formed structures (Rissanou *et al.*, 2013 ▸). The di­phenyl­alanine dipeptide crystallizes in the hexa­gonal space group *P*6_1_ (Görbitz, 2001 ▸) and forms hollow nanotubes with hexa­gonal symmetry that can be used as molds for inorganic nanowires (Reches & Gazit, 2003 ▸) and which display a series of inter­esting physical properties (Rosenman *et al.*, 2011 ▸). Despite phenyl­alanine-rich peptides, especially di­phenyl­alanine (Phe–Phe), being the most studied of the aromatic category (Reches & Gazit, 2006*a* ▸,*b* ▸), due to their impressive condition-dependent array of self-assembling formations, aliphatic peptides or mixed aromatic–aliphatic dipeptides seem just as promising, exhibiting various adopted conformations ranging from amyloid fibrils to microtubes. Recently, a detailed investigation of the structural and conformational properties of the unprotected alanine–isoleucine (Ala–Ile) and isoleucine–isoleucine (Ile–Ile) dipeptides in aqueous solutions using a combination of all-atom molecular dynamics (MD) simulations and experiments showed that the Ala–Ile dipeptide can form microtubes with hexa­gonal symmetry upon evaporation on glass surfaces (Rissanou *et al.*, 2020 ▸). This dipeptide also crystallizes in the hexa­gonal space group *P*6_1_ (Görbitz, 2003 ▸). The Leu–Leu dipeptide crystallizes in the ortho­rhom­bic space group *P*2_1_2_1_2_1_ (Görbitz, 2001 ▸) and a report has appeared showing the formation of nanotubes with ortho­rhom­bic sym­metry (Handelman *et al.*, 2016 ▸). It seems, therefore, that a correlation exists between the space group of crystallization and the geometrical features of the formed nano- or microtubes. Of note, the hollow peptide nanotubes, regardless of their symmetry, display very inter­esting optical properties with applications ranging from waveguiding to biochips (Apter *et al.*, 2018 ▸).

Protecting groups, especially at the N-termini, such as *t*-Boc (*tert*-but­oxy­carbon­yl), Fmoc (fluorenyl­meth­oxy­carbon­yl) and Z [*N*-(benzyloxycarbonyl)], influence the outcome of the self-assembly process. For example, the Fmoc group is an excellent gelator moiety and the Fmoc-Phe–Phe peptides form hydro­gels that support cell attachment and proliferation and can be used as scaffolds in tissue engineering applications (Jayawarna *et al.*, 2006 ▸; Orbach *et al.*, 2009 ▸). The Z-Phe–Phe-protected dipeptide, when dis­solved in various solvents and at various concentrations, can form a variety of nanostructures, including nanowires, fibers and nanospheres (Brown *et al.*, 2018 ▸). Moreover, in a two-solvent system, hydro­gels are formed when the water content exceeds 75%. Crystals were grown by slow evaporation from methanol and X-ray crystallography revealed a structure arrangement with a space group symmetry of *P*2_1_2_1_2_1_. No microtubes were reported among the adopted nanostructures (Brown *et al.*, 2018 ▸). 
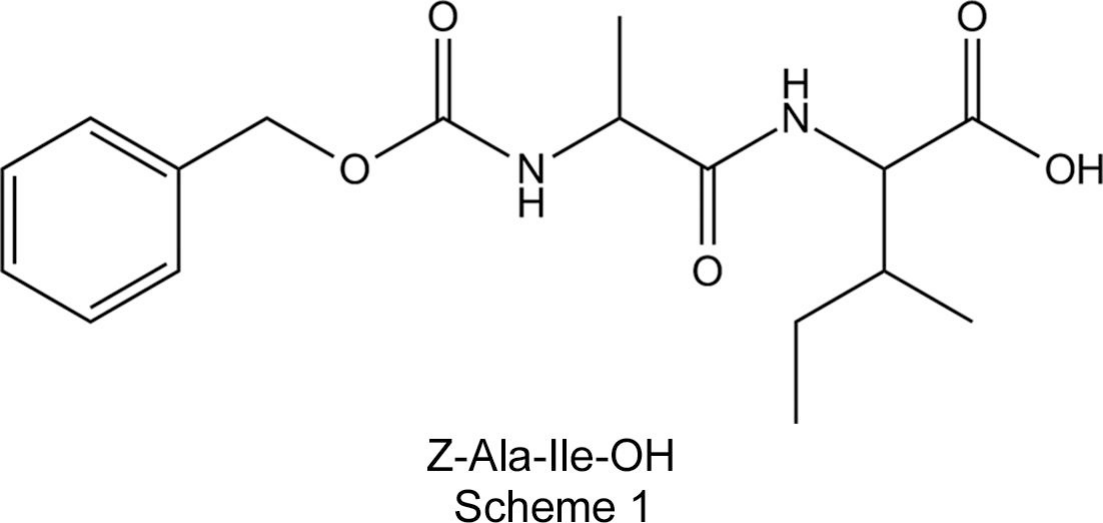


**Figure 1:**
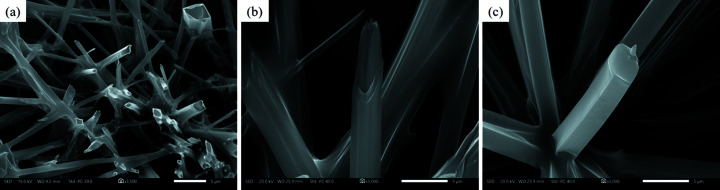
FESEM images of the Z-Ala–Ile microtube assemblies after incubation in aqueous solution for 24 h and subsequent deposition on a glass slide. (*a*) Hollow microtubes emanate from the surface, (*b*) higher resolution image of a hollow microtube tip shows a layered substructure and (*c*) a view of a full microtube.

Here we focus on the crystal structure determination and the microstructure formation of an aliphatic dipeptide, ala­nine–isoleucine, protected with a Z group at the N-terminus. The protected dipeptide crystallizes in the ortho­rhom­bic space group *P*2_1_2_1_2_1_ and forms hollow microtubes with ortho­rhom­bic symmetry upon evaporation on the surfaces, as shown by field emission scanning electron microscopy (FESEM) (Fig. 1 ▸). The change in the single-crystal structure com­pared to that adopted by the unprotected dipeptide, as well as the change in the symmetry of the self-assembled microtubes, illustrate the point that protecting groups can enable the generation of microtubes with a different symmetry from that of the original dipeptide building block.

## Experimental

Z-(l)Ala–(l)Ile-OH [*N*-(benzyl­oxycarbon­yl)-l-alanyl-l-iso­leu­cine] was purchased from Bachem (Bubendorf, Switzerland) in the form of the lyophilized powder.

### Microstructure formation

The peptide powder was dissolved in ultrapure water with the alternating use of an ultrasonic water bath and strong vortex agitation. More specifically, the peptide–water-containing vial was initially transferred to the ultrasonic water bath for a duration of 30 s, at a temperature of 55 °C, and vigorous vortexing was subsequently carried out. This process was repeated until com­plete dissolution of the peptide powder was observed. Finally, the solution was incubated for 24 h at room temperature. Aliquots of the samples prepared for microstructure formation (10 µl) were deposited onto glass slides and left to dry in air.

### FESEM observation

Dried samples were sputter-coated with a 15 nm thick film of gold or gold/palladium (Baltec SCD 050) and imaged using a Field Emission Scanning Electron Microscope (JEOL IT700HRJ) operating at 15 and 20 kV. These experiments were conducted at the Electron Microscopy Facility at the Department of Biology of the University of Crete.

### Crystallization

The peptide powder was disolved in water (*T* = 55° C) at a concentration of 1.34 mg ml^−1^ under permanent sonication in a waterbath. 100 µl drops were set up directly on siliconized glass cover slides (Hampton Research), which were placed inside plastic Petri dishes that were left open, partially open and closed in a room regulated at 19–20 °C, where evaporation took place. The best crystals were obtained in partially open and/or closed plates after overnight to 2 d of evaporation.

### Measurement

Single needle-shaped crystals of approximately 200–300 µm length were mounted on cryoloops fixed at their base by a tiny amount of silicone vacuum grease. Three data sets were collected from three different crystals at the ESRF microfocus automated beamline ID30B (McCarthy *et al.*, 2018 ▸) equipped with an MD2-S micro­diffractometer, a FlexHCD sample chan­ger and an Eiger2 X 6M detector (Dectris). The data collection hardware was configured with a canted undulator of −2.2 mrad, an Si[111] monochromator, secondary slits and a compound refractive lens (CRL) transfocator for vertical focusing. All three data sets were collected with very high redundancy, total transmissions of 10 (for two data sets) and 38.4% (for one data set), and a starting flux between 3.74 e+12 and 1.85 e+13 ph s^−1^. For the three individual data sets, the space group and processing were calculated automatically by the different software pipelines available in the beamline [*XDSAPP* (Krug *et al.*, 2012 ▸) and *XIA2-DIALS* (Winn *et al.*, 2011 ▸)]. However, data collections were integrated again manually using *XDS*, merged with *XSCALE* (Kabsch, 2010 ▸) and scaled with *AIMLESS* (Evans & Murshudov, 2013 ▸).

### Structure solution and refinement

All non-H atoms were detected as the highest peaks with the TREF (direct methods with tangent formula phase refinement) option in *SHELXS* (Sheldrick, 2008 ▸). Anisotropic refinement gave an *R* value of 0.0661 and an *R*_free_ value of 0.0816 for all data. All H atoms were located as the highest peaks in difference Fourier syntheses and refined freely. Due to their unlikely geometry and unreliable displacement parameters, 21 H atoms were included at a later stage in the refinement with riding-atom positions and riding displacement parameters associated with the attached non-H-atom, while for the three H atoms participating in hydrogen bonding, the atom positions were included with the riding model and the displacement parameters were refined freely.

## Results and discussion

**Figure 2:**
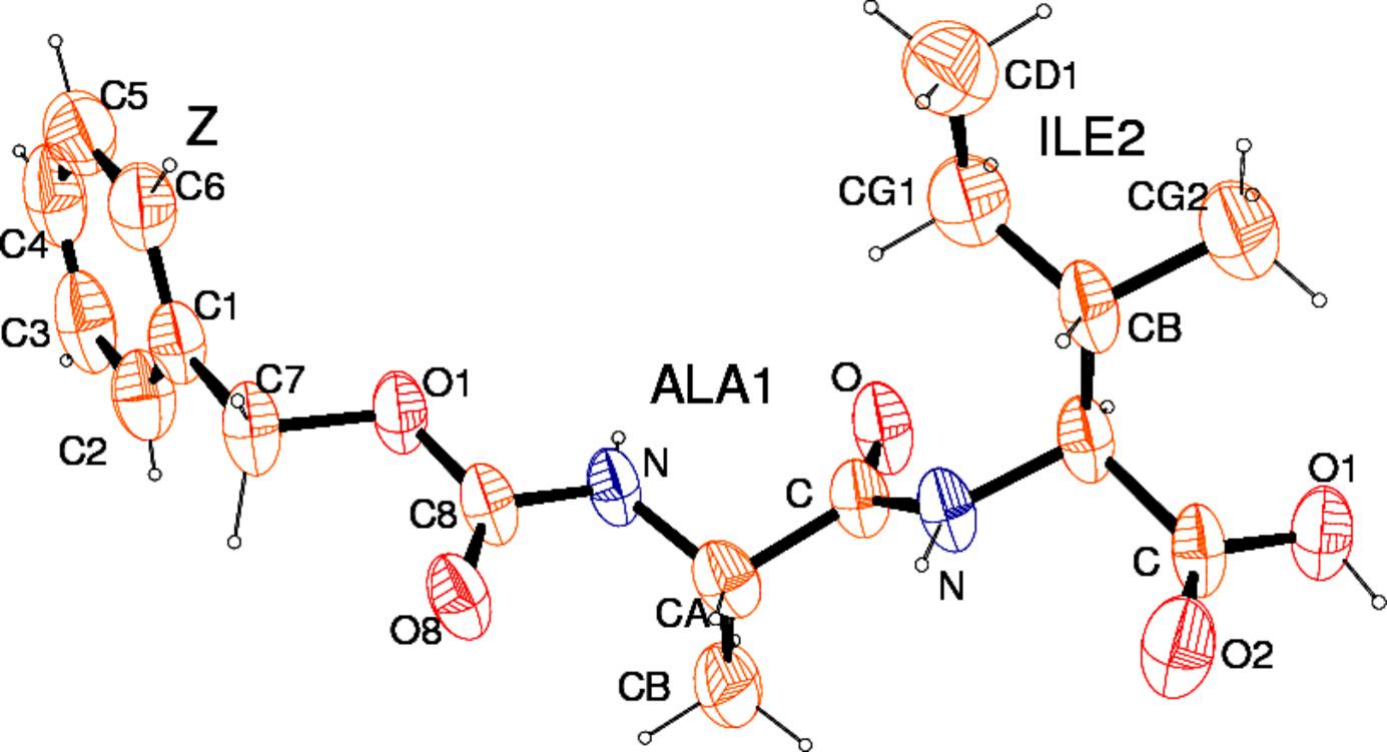
The mol­ecular structure of Z-Ala–Ile-OH, showing 50% probability displacement ellipsoids (Farrugia, 2012 ▸). For clarity, the residue abbreviations Z, Ala and Ile are also included.

After incubation of Z-Ala–Ile in aqueous solutions for 24 h and deposition on glass slides, FESEM images showed hollow microtubes emanating from the surface with openings ranging from the micrometer to the submicrometer range [Fig. 1 ▸(*a*)]. Higher magnification images revealed layered substructures at the microtube tips [Fig. 1 ▸(*b*)]. Filled microtubes with crystal edges were also observed with dimensions similar to the hollow microtubes [Fig. 1 ▸(*c*)]. In the mol­ecular structure of Z-Ala–Ile (Fig. 2 ▸), Ala1 adopts an extended conformation, with torsion angles of φ = −145.1 (1)° and ψ = 123.2 (5)° (Richardson & Richardson, 1989 ▸). Ile2 also adopts an extended conformation, with torsion angles of φ = −133.4 (5)° and ψ = 144.6 (4)°, by taking into account the fourth atom as O1 of the C-terminal OH group, and torsion angles of φ = −133.4 (5)° and ψ = −35.4 (7)° with O2, the carbonyl oxygen, as the fourth atom. The extended conformations adopted by Ala1 and Ile2 diverge significantly from the fully extended conformation (φ,ψ) = (−180°,180°) and lie in the energetically preferred region of the Ramachandran plot for proteins in β-strands (Voet & Voet, 2004 ▸).

**Figure 3:**
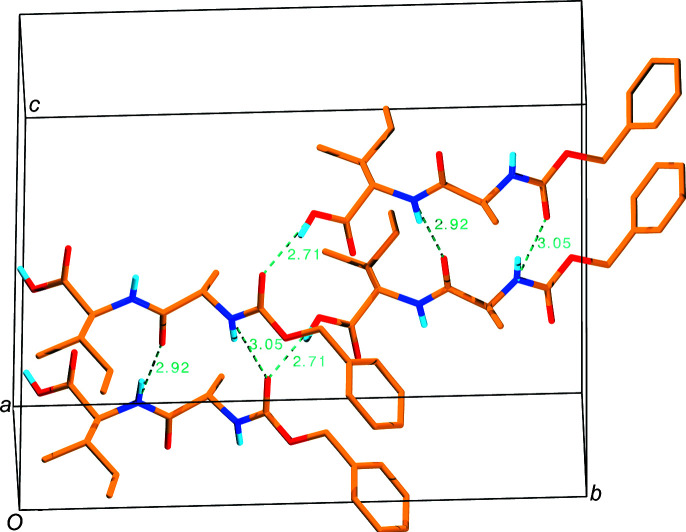
Hydrogen bonding of Z-Ala–Ile-OH, viewed approximately along the *a* axis. Hydrogen bonds are shown as dashed lines in cyan with the donor–acceptor distances (*D*⋯*A*) in Å.

The assembling of the symmetry-related mol­ecules is shown in Fig. 3 ▸. Two mol­ecules [symmetry codes (*x*, *y*, *z*) and (−*x*, *y* + 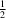 , −*z* + 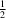 )], along with their *a*-axis-translated counterparts, show the network of hydrogen bonds. There is a tail-to-head hydrogen bond between the OH group of Ile and the carboxyl group of the Z-protection group of the symmetry-related mol­ecule, with a distance of 2.713 (5) Å, while along the *a* axis, two mol­ecules are hydrogen bonded twice, namely, N [Ala; at (*x* + 1, *y*, *z*)] to the carboxyl group of the Z-protection group [at (*x*, *y*, *z*)], with a distance of 3.049 (6) Å, and N [Ile; at (*x*, *y*, *z*)] to O [Ala; at (*x* + 1, *y*, *z*)], with a distance of 2.915 (6) Å. Thus, planes of hydrogen-bonded mol­ecules are formed parallel to the *ab* plane.

**Figure 4:**
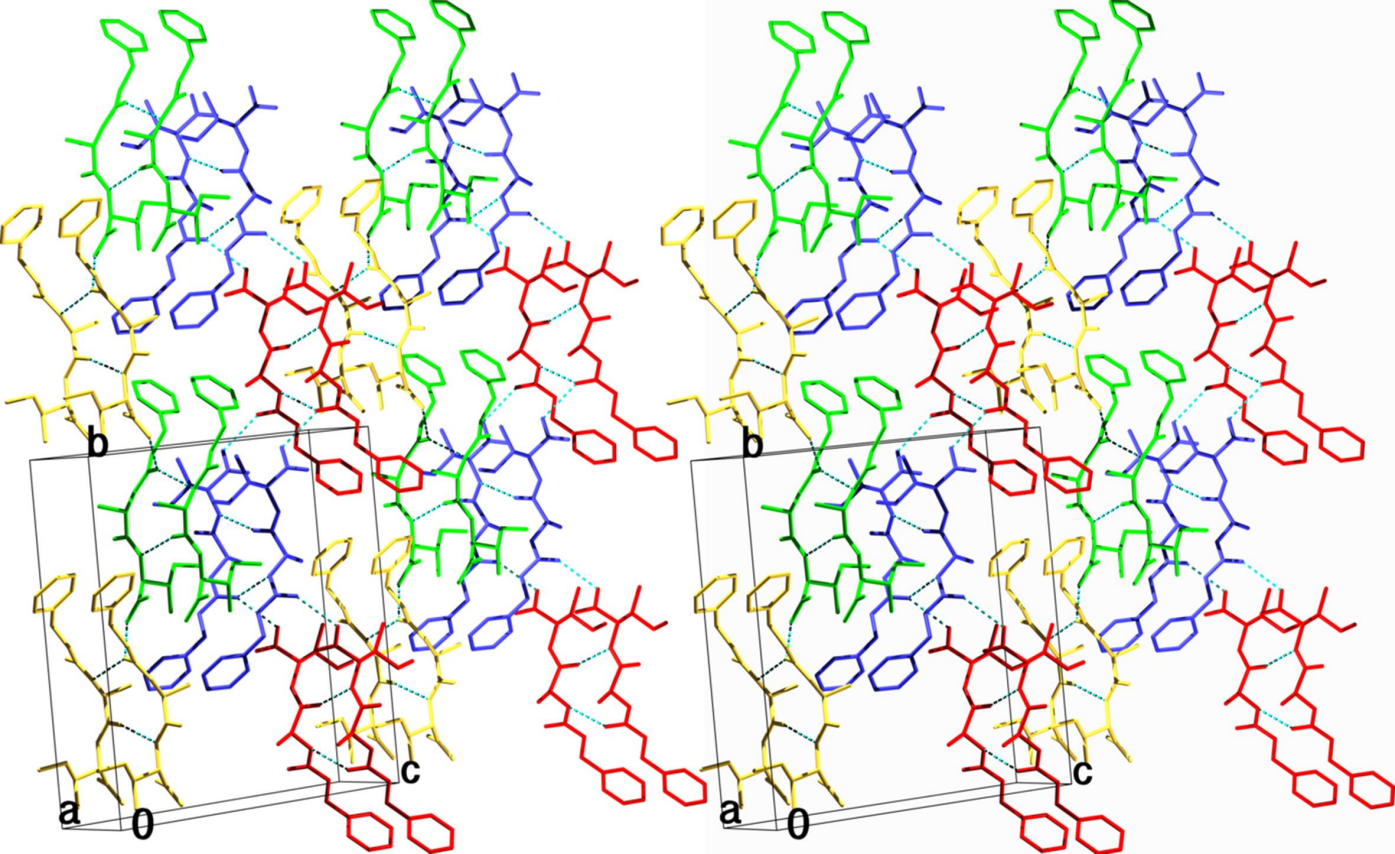
Wall-eyed stereoview of the crystal packing of Z-Ala–Ile-OH, viewed approximately along the *a* axis. Different colours denote mol­ecules related by space-group symmetry. Hydrogen bonds are shown as dashed lines in cyan.

Fig. 4 ▸ shows the packing in the crystal. 16 mol­ecules of four unit cells are shown twice, translated along the short *a* axis, thus showing a 2 × 2 × 2 assembly of mol­ecules. Different symmetry equivalents are shown in different colours. Yellow (*x*, *y*, *z*) and green (−*x*, *y* + 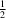 , −*z* + 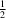 ) are tail-to-head hydrogen bonded, and form horizontal planes parallel to the *ab* plane. In the same way, blue (−*x* + 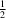 , −*y*, *z* + 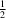 ) and red (*x* + 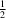 , −*y* + 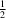 , −*z*) mol­ecules also form horizontal planes parallel to the *ab* plane. The yellow–green and the anti­parallel blue–red planes stack together *via* hydro­phobic inter­actions. Equivalent arene-ring atoms of the Z-protection groups of the same colour have an *a* axis distance of 4.820 (1) Å, and the shortest distance between these parallel neighbouring arene-ring atoms is 3.541 (9) Å, with parallel π–π stacking. In contrast, the arene rings of the Z-protection groups of the non-hydrogen-bonded green-and-blue and yellow-and-red mol­ecules have an angle of about 90° (in Fig. 4 ▸, the green and blue are above, and also the yellow and red are above in the middle of the figure), with a closest distance of 3.644 (9) Å between the C atoms of neighbouring rings. In this way, a C—H⋯π attractive force (Janiak, 2000 ▸) exists in the *b* direction between the non-hydrogen-bonded planes. Since one ring has this attractive force with two rings of symmetry-related mol­ecules which are translated along the small *a* axis, this force acts also along the smallest crystal axis *a*. All these inter­actions between the Z-pro­tection groups might stabilize further the crystal formation and increase stability. The presence of the Z-pro­tection group in the Z-Ala–Ile dipeptide would also influence the three-dimensional arrangement observed in the Z-Ala–Ile dipeptide microtubes which differ in com­parison to the arrangement observed by scanning electron microscopy (SEM) for the unprotected Ala–Ile dipeptide where hexa­meric microstructures were observed under similar conditions (Rissanou *et al.*, 2020 ▸). This observation shows that the pro­tection groups might have a strong influence on the self-assembly of dipeptides and could be used to introduce a new level of controlled variability. In addition, the *ab* planes stack together in the *c* direction *via* apolar contacts in such a way that the bulky Ile side chains fit in the bent backbone of the peptide. These inter­actions enhance a close packing of the mol­ecules, which is reflected by the relatively high crystal density of 1.258 Mg m^−3^ in com­parison with other peptide structures without cocrystallized solvent mol­ecules. A relationship between close packing, high crystal density and suitable crystal size for X-ray analysis was observed recently (Gessmann *et al.*, 2020 ▸). A search for related structures and their ability to form nanoparticles in the Cambridge Structural Database (CSD; Groom *et al.*, 2016 ▸) revealed the Ala–Gly-OH, Ala–Ala-OH and Ala–Val-OH di­peptides (Houton *et al.*, 2012 ▸). The five available structures are N-terminal protected with a naphthalene group and the connection to the peptide is attached either at position 1 or 2 of the naphthalene double-ring system. The different peptides form crystals, clear gels or turbid gels. The naphthalene-protected Ala–Gly-OH and Ala–Ala-OH dipeptides form ortho­rhom­bic crystals, while the naphthalene-protected Ala–Val-OH dipeptide crystals are monoclinic. The backbone conformation (from the C=O group of the protection group to the C-terminal C atom) com­prises nine main-chain atoms and is similar to the backbone conformation of the herein described Z-Ala–Ile-OH dipeptide, with average r.m.s. deviations of less than 1 Å. Crystals and microtubes are produced in almost the same way. The two smaller dimensions of the single crystal used for X-ray analysis are about 0.05 mm or 50 µm (Table 1 ▸), and the rectangular basis of the microtubes is about 0.8 µm × 0.8 µm to 2 µm × 2 µm [Fig. 1 ▸(*a*)]. Thus, the dimensions differ by factors of only about 62 to 25, pointing to a similar way of growing. One could speculate that the microtubes have the same packing as the crystal and that the ‘walls’ of the microtube should be the hydrogen-bonded crystal dimensions *a* and *b*. Also, the shape of the crystal needles resembles the shape of the microtubes, suggesting that a multi-step hierarchically oriented crystallization model might be operating, where the basic mol­ecular building blocks self-assemble, as a result of attractive depletion and entropic ordering, into primary bundles with the subsequent emergence of hollow tubes with crystal faces following a final conversion into crystals as proposed (Yuan *et al.*, 2019 ▸). Although the exact mechanism that relates the peptide building block structure to the microtubular assemblies remains to be elucidated, it appears that at least a correlation can be established between the highly ordered multilayer substructures and the final crystal packing and structure. Elucidation of the exact mechanisms in the future may enable the rational design of mol­ecular building blocks that could self-assemble into predictable supra­molecular geometries and expand the library of available building blocks for nanotechnological applications.

## Supplementary Material

Crystal structure: contains datablock(s) I, global. DOI: 10.1107/S2053229623004849/op3019sup1.cif

Structure factors: contains datablock(s) I. DOI: 10.1107/S2053229623004849/op3019Isup2.hkl

CCDC reference: 2211673

## Figures and Tables

**Table 1:** Experimental details

Crystal data	
Chemical formula	C_17_H_24_N_2_O_5_
*M*_r_	336.38
Crystal system, space group	Orthorhombic, *P*2_1_2_1_2_1_
Temperature (K)	100
*a*, *b*, *c* (Å)	4.820 (1), 19.110 (4), 19.280 (4)
*V* (Å^3^)	1775.9 (6)
*Z*	4
Radiation type	Synchrotron, λ = 0.97625 Å
μ (mm^−1^)	0.2
Crystal size (mm)	0.2 × 0.05 × 0.05

Data collection	
Diffractometer	ESRF beamline ID30B
No. of measured, independent and observed [*I* > 2σ(*I*)] reflections	22019, 1887, 1663
*R*_int_	0.154
θ_max_ (°)	30.5
(sin θ/λ)_max_ (Å^−1^)	0.520

Refinement	
*R*[*F* ^2^ > 2σ(*F* ^2^)], *wR*(*F* ^2^), *S*	0.060, 0.174, 1.10
No. of reflections	1887
No. of parameters	221
H-atom treatment	H atoms treated by a mixture of independent and constrained refinement
Δρ_max_, Δρ_min_ (e Å^−3^)	0.30, −0.28
Absolute structure	Set to match the known absolute configuration of Z-(L)Ala–(L)Ile-OH

Computer programs: *AIMLESS* (Evans & Murshudov, 2013 ▸), *SHELXS86* (Sheldrick, 2008 ▸), *SHELXL2014* (Sheldrick, 2015 ▸), *COOT* (Emsley *et al.*, 2010 ▸), *SwissPDBViewer* (Guex & Peitsch, 1997 ▸), *CHEMDRAW* (Mills, 2006 ▸), *ORTEPIII* (Burnett & Johnson, 1996 ▸), *ORTEP-3 for Windows* (Farrugia, 2012 ▸) and *POV-RAY* (Cason, 2004 ▸).
